# GenoSets: Visual Analytic Methods for Comparative Genomics

**DOI:** 10.1371/journal.pone.0046401

**Published:** 2012-10-03

**Authors:** Aurora A. Cain, Robert Kosara, Cynthia J. Gibas

**Affiliations:** 1 Department of Bioinformatics and Genomics, The University of North Carolina at Charlotte, Charlotte, North Carolina, United States of America; 2 Department of Computer Science, The University of North Carolina at Charlotte, Charlotte, North Carolina, United States of America; CSIR-Institute of Microbial Technology, India

## Abstract

Many important questions in biology are, fundamentally, comparative, and this extends to our analysis of a growing number of sequenced genomes. Existing genomic analysis tools are often organized around literal views of genomes as linear strings. Even when information is highly condensed, these views grow cumbersome as larger numbers of genomes are added. Data aggregation and summarization methods from the field of visual analytics can provide abstracted comparative views, suitable for sifting large multi-genome datasets to identify critical similarities and differences. We introduce a software system for visual analysis of comparative genomics data. The system automates the process of data integration, and provides the analysis platform to identify and explore features of interest within these large datasets. GenoSets borrows techniques from business intelligence and visual analytics to provide a rich interface of interactive visualizations supported by a multi-dimensional data warehouse. In GenoSets, visual analytic approaches are used to enable querying based on orthology, functional assignment, and taxonomic or user-defined groupings of genomes. GenoSets links this information together with coordinated, interactive visualizations for both detailed and high-level categorical analysis of summarized data. GenoSets has been designed to simplify the exploration of multiple genome datasets and to facilitate reasoning about genomic comparisons. Case examples are included showing the use of this system in the analysis of 12 *Brucella* genomes. GenoSets software and the case study dataset are freely available at http://genosets.uncc.edu. We demonstrate that the integration of genomic data using a coordinated multiple view approach can simplify the exploration of large comparative genomic data sets, and facilitate reasoning about comparisons and features of interest.

## Introduction

### Background

To make sense of genomic sequence data, genomes are annotated with information that can include results from the application of computational tools and from laboratory experiments. This layered set of information describes the location of features, their similarities with other known features, and their functional and contextual properties. A typical workflow in the comparative analysis of microbial genomes begins with developing feature annotations. The location of genes and other features is established based on the existence of a transcript, or predicted using *ab initio* algorithms. A feature’s function can often be inferred correctly from high-confidence sequence relationships to other features with known function, even if experimental evidence for the function is not available. Therefore a comparative analysis system must support two major types of operations: defining regions on a single genome based on some property or content information (annotative operations), and defining relationships between regions in one or more genomes based on a comparative analysis (comparative operations).

Comparative analysis of genome sequence can involve anything from pairwise alignment of complete genome sequences, direct comparison of the sequence, or comparison of number and order of individual genes in the genome. With the growing number of available genomes, what we really want to be able to do to facilitate understanding of genomic differences is to compare many genomes, cluster their genes into putative ortholog clusters, and identify common and differentiating features at different levels of taxonomy. The concept of a pan-genome was first introduced by Medini in 2005 [1] and is defined as the entire gene vocabulary used by a given taxonomy group. A pan-genome can be defined at the species level, but groupings at higher taxonomic levels are also of interest. The core genome of a taxonomy group is the set of genes that are shared by all species or strains, and the dispensable genome is the set of genes unique to one or to a subset of species within the group. Comparing these gene sets at different levels of taxonomy provides insight into pathogenicity, functional potential, and genes required for different environmental niches.

These comparative genomics questions are fundamentally set-based queries, in which the scientist is asking to see only the features that meet specific criteria – for example, only features which are unique to genome A, belong to a particular functional category, and have experimental support for the existence of the gene. Genes belonging to the desired category have membership in three independently determined sets, which may intersect. To execute such a query starting from scratch, a biologist would have to run several different programs, extract annotations from multiple files, and then compare gene lists to determine which genes met all of the specified criteria. Of course, construction and use of a database makes such queries possible, but the matter of translating a question involving multiple set-based criteria to a workable SQL query still leaves the user with a learning curve to climb. The GenoSets system is an experiment in using visual analytic methods to enable the user to intuitively subdivide a large, multi-genome dataset based on orthology, taxonomic criteria, and functional criteria. Linked visualizations provide the means to access different categories in the dataset and to make selections to define the query using only a series of mouse clicks.

### Current Approaches to Integrated Querying

Users of set-based queries (whether they are aware of the set-nature of their questions or not) are not served particularly well by linear browsers, which are focused on providing an integrated detail view when the user has already identified a gene or coordinate range of interest. For example, the UCSC Genome Browser [2], [3] makes it possible to add comparative information as layers of tracks that display the alignments to a gene, and display these together with associated annotation information. This type of display does not allow the user to easily analyze categories of genes that span multiple, physically separated genomic regions. And as the number of genomes that are being compared increases, the number of annotation tracks explodes into a view that can be hard to interpret.

GenoSets offers an alternate visual summarization paradigm. Instead of creating new ways to compress the linear genome view, we are creating ways to summarize and then subdivide the descriptive data associated with a genome. We can summarize the gene content independently of position in the genome, and allow the user to formulate the kind of high-level questions that biologists often ask of their layered, complex genome data: what differentiates genome A from genome B and genome C? What differentiates one version of the annotation from another? How is the gene content different?

Script-based data-mining approaches or direct interaction with databases via SQL queries have generally been used for set-based searches, and recently some of the major browser-driven genome resources have added web-based, menu-driven data mining tools that can build these queries. The web tool Galaxy [4] has been created to work with the UCSC Genome Browser. Galaxy uses the intersection of track information to categorize genes. Similarly, GenePhony [5] is a tool that allows the user to construct queries as the intersection of multiple sets. However, in both cases, many steps of data manipulation are required both within and outside the interface to achieve the desired result, and so these tools are not particularly friendly to users untrained in bioinformatics, or to those unfamiliar with the structure of the underlying database.

To illustrate the utility of the GenoSets system, we present two case studies using the genus *Brucella*, which we have previously analyzed [6], [7]. The *Brucella* genus is interesting because of variable host preferences and symptoms of infection among species that appear quite closely related at the gene content level. Although *Brucella* species typically infect livestock animals there have also been many fatal cases of brucellosis in humans. Only certain species of *Brucella* are capable of causing human infection, and some of the species have no reported cases of human infection [8]. GenoSets allows the user to group genomes based on taxonomic groupings or on phenotypes of interest. We demonstrate in the case studies that this feature facilitates identification of significant genomic differences.

## Methods

### Data

GenoSets currently supports annotation parsing, to establish the content of the genome, ortholog clustering, to establish consistent gene definitions across an entire set of genomes, and Gene Ontology (GO) term assignment, to provide a means for further categorizing gene content based on apparent function.

Genomic annotations can be automatically downloaded from the EMBL-Bank [9] public repositories or uploaded in GFF format. Genomes can be selected from a list of completed microbial projects, or by EMBL accession identifier, or by upload of a custom file for a genome that is not already published. See Table S1 for a complete list of accessions for the genomes used in this analysis. All the details of the annotation files are parsed and stored in the GenoSets database. The minimal description of a feature in the database includes the genomic coordinates (start and end positions and the strand) and textual descriptions of the feature. Additional details such as product description, protein domains, and experimental notes if they exist, are stored in the database. Taxonomy information for published genomes can be accessed via links out to NCBI’s Taxonomy, and the system allows upload of a custom phylogenetic tree by the user. User-provided genome data can also be imported into the system for comparison with published genomes.

Establishing orthology relationships among genomes in the database is required for queries based on the presence or absence of genes. Orthology relationships across a set of genomes can be estimated using grouping techniques based on sequence similarity. Some of the best known methods include the clustering algorithm underlying the Clusters of Orthologous Groups (COG) database [10], Inparanoid [11], and OrthoMCL [12]. We chose to use OrthoMCL [12], which uses a Markov Cluster algorithm to group putative homologs based on sequence similarity, as the primary ortholog clustering method in GenoSets. OrthoMCL has been shown to outperform other stand-alone methods for ortholog clustering [13]. The OrthoMCL software is run from within GenoSets. A wizard allows the user to define optional parameters, and stores the parameters along with clustering results in the GenoSets database.

To provide a standard means of comparison for feature attributes, GenoSets uses the Gene Ontology (GO) as a structured controlled vocabulary [14]. These terms can be applied to genes in multiple genomes and at multiple levels of granularity. Several public databases exist that have GO term assignments to genes either through manual or automated curation [15]–[17]. GenoSets currently supports the download of GO annotations from the EMBL GOA database [15]. The user needs only to start the process and the annotations are automatically downloaded.

Although the Gene Ontology provides a standardized vocabulary and can summarize genes at multiple levels of granularity, the resulting set of GO annotations for multiple genomes can include hundreds or thousands of GO term associations. Gene Ontology term enrichment analysis identifies GO terms that are significantly overrepresented or underrepresented within a subset of genes. Currently, we support GO term enrichment analysis using the software program, Ontologizer [18] which is run directly from the GenoSets system. Ontologizer optionally offers several methods for GO term enrichment analysis including a parent-child method (Grossman, 2007), topology based methods (Alexa, 2006), and a model-based method (Bauer, 2010), all of which serve to minimize correlation biases due to the hierarchical structure of the ontology. Additionally, Ontologizer offers several procedures for multiple-testing correction including Bonferroni and resampling-based Westfall-Young (Westfall 1993) corrections. The user is able to select these options and optionally define the population set used in each analysis.

The enrichment analysis uses the annotations associated with each ortholog cluster, meaning if a gene was annotated with a specific GO term, then all the genes in that cluster are also associated with that GO term.

Each of the major data operations – annotation of individual genomes, clustering of orthologs, association of ontology terms and enrichment analysis – is required for set-based querying, but the choice of methods at each step is flexible and the system supports substitution of pipeline components as available methods evolve.

### Gene Set Visualizations

The primary visualization for set-based queries is the Parallel Sets view. Parallel Sets [19], [20] is a visual method designed for analysis of data from large collections of discrete entries. Although originally developed for application to demographic and customer survey data, GenoSets uses this technique as the controlling visualization through which the user can define sets of genes and features.

The central idea behind this visualization is the use of subsets that are defined by combinations of categories. Like a Venn Diagram, the Parallel Sets view defines set membership in terms of inclusion and exclusion in a category. Each categorical dimension is represented by a horizontal bar. As dimensions are added to the display, the bars are stacked vertically. The bars representing an individual dimension are connected to one another with ribbons. The width of the connecting ribbons represents the number of items that are in the connecting categories of the dimensions. Therefore, ribbons represent set intersections across multiple criteria, and the width of the ribbon represents the number of items that match those criteria, or in other terms, the frequency distributions of the categories within that dimension.

**Figure 1:**
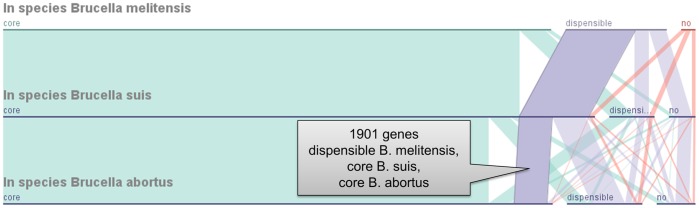
Parallel Sets for categorical partitioning of gene sets. A Parallel Sets view is used to subdivide genes that occur in genomes of the Brucella genus. The highlighted ribbon contains the genes that are part of the dispensable gene vocabulary of known B. melitensis species, but appear to be part of the common core gene set in B. suis and B. abortus species. When selected, the list of genes attached to the ribbon propagates to other coordinated views.

The Parallel Sets view in GenoSets shows the number of genes that are members of the different categories displayed. Each horizontal axis represents the content of a single genome, or of a grouping based on function or taxonomy. The width of a ribbon represents the number of genes in each category. Figure 1 shows GenoSets being used to subdivide the *Brucella* data. In this view, we are highlighting genes that are part of the core genome of known *B. suis* and *B. abortus* species, while being dispensable in *B. melitensis*. The first dimension, “in species *Brucella melitensis*”, has three categories: core, dispensable, and no. We have multiple strains of each of these three species, so that term “core” means that all *B. melitensis* strains analyzed have those genes, “dispensable” means only some have those genes, and “no” means that none of them have those genes. The highlighted ribbon in Figure 1 leads the user to a group of 1901 genes that are dispensable in *B. melitensis* but are core to both *B. suis* and *B. abortus*. This example shows the use of the Parallel Sets view to show nine discrete categories simultaneously, many more than is possible with the familiar Venn Diagram. With the summary view that Parallel Sets provides, users are able to see not only the gene count but also the relationships between categories. Case study 2 further demonstrates the use of Parallel Sets and the other connecting visualizations to identify features of interest based on the visual cues the displays provide.

**Figure 2:**
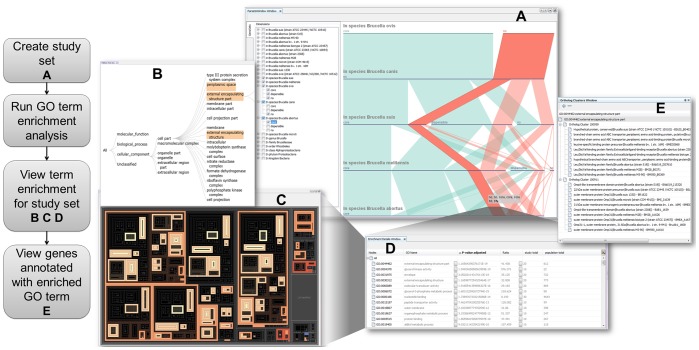
Overview of the GenoSets user interface. The multiple views allow the user to progressively build queries at multiple levels of detail. The Parallel Sets view (A) allows you to create study sets by partition your genes based on orthologous relationships across multiple taxonomic levels. The study sets created in (A) can then be viewed along with associated GO enrichment in the Tree Navigator view (B), Treemap view (C), and the Enrichment Details view (D). Each of these views is displaying the same information in alternative forms for a better overall analysis. The user may further filter the dataset by selecting a GO term from views B, C, or D. The details for each gene associated with the selected GO term are displayed in the Ortholog Cluster view (E). A demonstration video of this case study can be found at http://genosets.uncc.edu.

The core analytical visualization implemented in the system is the Parallel Sets view. However, additional visualizations are implemented to allow the user to explore and sort data in a collection where hundreds of thousands of genome features must be manipulated simultaneously. We have implemented Parallel Sets along with additional views in a Coordinated Multiple Views (CMV) interface. Figure 2 illustrates the complete interface. In a CMV system, all visualizations have the ability to communicate with one another, meaning that selections in one view are propagated to all others. Within the interface, the user can navigate the dimensions of the dataset and get instant feedback on the selections. The user can select a subset of data in one display and then create another view from that selected subset. For example, the user may select the genes in one genome that do not have orthologs in another genome, then propagates that set to a view that displays the GO term enrichment associated with those genes.

Study sets are built by making selections in the Parallel Sets view. The study sets created can be viewed and managed from the Study Set Navigator. The study set navigator allows the user to rename study sets and view the categories that were used to create them. All of the sets in this list can be analyzed for GO term enrichment and can be added or removed at any time. This view is also connected to the CMV interface, and all the views showing details about a specific study set are updated when the user selects a set in this navigator.

### Hierarchy Visualizations

The GO Tree Explorer (Figure 2B) and the GO Treemap (Figure 2C) both represent the results obtained from a GO Term enrichment analysis. The GO Tree Explorer view (Figure 2B) is a view of the hierarchy of terms in a classification drawn as a traditional tree visualization; The GO Treemap and the GO Tree Explorer work together to help the user interpret the levels of the GO hierarchy. All selections in these views are coordinated together such that a selection in one updates what is viewed in the other.

The GO Treemap view (Figure 2C) is a visual representation of the results obtained from a GO term enrichment analysis. It represents the hierarchical structure of the Gene Ontology together with the categorical information about the number of genes associated with each term and the statistical significance of the term. Treemaps have been developed to simultaneously visualize both hierarchical and quantitative data [21] and have previously been used to summarize Gene Ontology terms, although not in the context of genome comparison. The GO Treemap displays the GO term hierarchy as nested rectangles with each GO term drawn as a single rectangle and all child GO terms are drawn inside the parent rectangle. The size of each rectangle represents the number of genes classified with that GO annotation in the entire population set [21]. In GenoSets, the treemap view is coordinated to previous set selections made by the user. When the user selects a study set, the GO Treemap is updated to represent the enrichment for that study set only. GO terms that have a significant p-value are highlighted. The contrasting highlight colors represent the ratio of the study set to population set for that specific term. If the term ratio is higher for the study set than the population set, the term is colored one color; otherwise, the term is colored the other contrasting color. The user is able to select the colors used in the display and also set the p-value cutoff threshold, which determines the range of values that are colored in the treemap.

In the GO Treemap view, as the user points to a GO term (represented as a rectangle) the display will show the name of the term, the number of genes annotated with that term, and the level in the GO hierarchy where the term is found. A search box allows the user to search for GO terms by name, and the display will highlight any matches containing the search word. The user can also interact with the visualization by selecting different study sets, and the treemap will update to show only terms enriched in the selected set. This allows the user to make direct comparisons between subsets, highlighting functional categories that may be enriched in one subset relative to another.

For both the GO TreeMap and GO Tree Explorer, the Gene Ontology is represented as a tree structure. However, the GO hierarchy is not a true tree structure, it is a directed acyclic graph in which parent nodes may have multiple child nodes and vice-versa, but contains no cycles (if the graph is traversed down the hierarchy, the starting node will be visited once and only once). Representing this as a tree structure creates some redundancy in the graph (i.e. if a child node has two parents, then that portion of the tree is shown twice).

The hierarchy visualization methods used to create the GO Treemap and Tree Explorer views are generalizable to other hierarchical data types; for instance, a hierarchical view could be connected to a taxonomic dimension in a larger dataset to allow the user to navigate through that hierarchy.

### Detail Views

Detail lists of gene information are available to the user from multiple points in the interface (Figures 2D and 2E).

Feature details are available from all of the views, and contain all of the known information about genes or features. The user can enhance feature detail views by uploading files created in other analyses; for example, a tab delimited file containing pathway information can be uploaded, and this information will then show in all the feature detail views. Right clicking on any item in the interface will show which details are available for that view.

The Enrichment Details (Figure 2D) view shows the results from the GO term enrichment analysis. It is in table format and includes the GO term identifier and name along with the p-value associated with that term for the selected set. The table also includes the study term total and population total which is the total genes in the study set and population set annotated with each GO term, respectively. The table also includes a ratio which is the total genes in the study set divided by the total genes in the population for each GO term. The table may be sorted by any of the displayed columns and also filtered by p-value ranges.

The Ortholog Cluster view (Figure 2E) lists genes that are members of any selected set, grouping each of the genes together by ortholog cluster into a list structure. The gene identifier, name, product description, and organism to which a gene belongs are all shown. Like all other views, this view is connected to the selections in Parallel Sets and in the hierarchical views. The list is filtered to show the genes in the selected study set and the selected GO term.

### User Interaction and Database Design

GenoSets is a flexible system that supports the set-based comparative analysis of an arbitrary collection of genomes chosen by the user, based on features defined by both annotative operations and comparative operations. One of the key components of the system is the ability to load data and perform calculations through wizards. GenoSets provides many wizards that step the user through downloading gene annotation files from the EMBL website, downloading Gene Ontology associations from GOA, and running ortholog clustering using OrthoMCL.

The creation of a new database is also performed using a wizard. When the user initiates a new database, the system creates it and all necessary tables. There is no need to run any database scripts or manual configurations; users need only to provide a user name and password with sufficient database privileges for the creation process. The user may also connect to an existing database through this wizard. Because the database can be housed either locally or on a remote server, multiple people can access the system simultaneously.

The database that supports GenoSets is a multi-dimensional data warehouse. The multi-dimensional design is a widely accepted approach for real-time data mining and knowledge discovery, allowing for rapid, ad hoc querying of large, dimensional datasets. This is typically the support database used in business intelligence software. The aim of business intelligence software is knowledge discovery with the ability to support aggregates and hierarchical relationships within data. Aggregation and drill-down functions summarize data within a dimension at varying levels of granularity within a hierarchy. The “slice and dice” functions allow a subset of the data to be selected based on criteria selected across multiple dimensions.

GenoSets uses a star schema model presented by Kimball [22]. In the star schema, source data is partitioned into facts, representing the numerical measurements and dimensions that give context to the facts. The associated textual information describing the fact is separated into dimensions. Dimensions can have a hierarchical structure which allows for the facts to be rolled up into aggregates, i.e. a member of a category at one level in the hierarchy will automatically be a member of its parent category at the next level up. In the GenoSets database, the central fact is the existence of a feature. A “measurement” of existence models that a feature exists and links it to the associated dimensions describing it. Because the fact is a measure of existence and not a numerical measure, aggregation by count is often the most logical summarization of the data.

Using this database design in the analysis of comparative genomic data allows for a comprehensive study of the relationships among multiple dimensions describing the data, and eliminates the need to examine each individual feature at its finest level of detail. It enables the identification of annotated features that meet a set of criteria that spans multiple dimensions. Combinations of dimensions that occur frequently together and rare combinations or outliers can easily be identified. A typical genomic query in microbial genomics can quickly be formulated as a multi-dimensional query, for example ‘What genes are members of the core genome of all *Brucella* species?’ or ‘what genes are members of the dispensable genome of *Brucella melitensis*, and are there functional classifications that are enriched among those dispensable genes?’.

**Figure 3:**
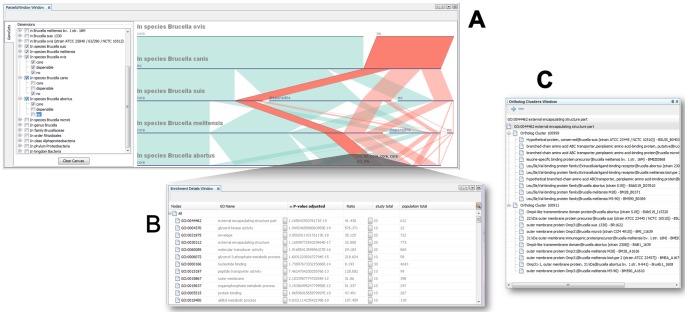
Using GenoSets to identify features in the highly pathogenic strains of Brucella. (A) The Parallel Sets view allows the user to create sets of genes that are only in the high-pathogenic strains. (B) Results of the GO enrichment analysis are shown in the Enrichment Details view. (C) View the genes associated with an enriched term in the Ortholog Cluster view. Genes are grouped together by ortholog clusters in this view.

## Results

To demonstrate the applicability of the GenoSets system for query and analysis of multi-genome datasets, we chose several sequenced genomes of species belonging to the genus *Brucella*. We have previously carried out a comparative analysis of the *Brucella* species [6], [23] using a predecessor to the GenoSets system, and identified regions useful for PCR-based species identification in a multi-step assay. The more recently sequenced genomes of the *Brucella* genus have since been analyzed and compared [8], [24], primarily with a focus on identifying pathogenicity islands. These previous studies provide us with a basis to evaluate the observations we can generate using GenoSets, and we demonstrate that the GenoSets process can be used to efficiently access insights that have generally been arrived at through a much more laborious manual analysis process.

**Figure 4:**
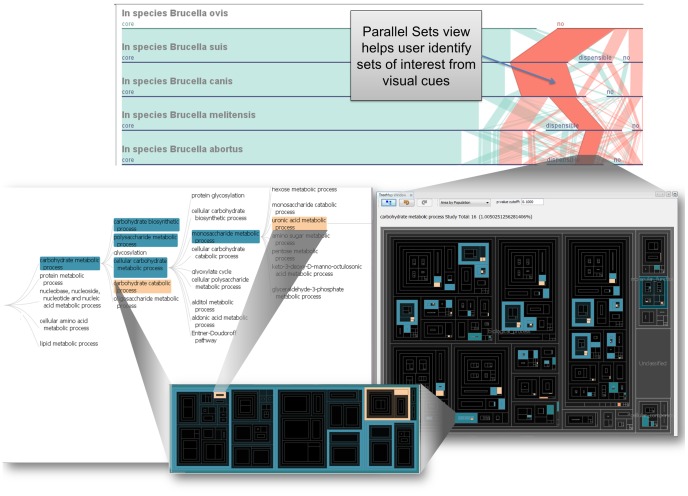
Application example of using GenoSets to identify key features of interest. (A) Parallel Sets highlights sets of interest that can be further analyzed using multiple alternative views. (B) GO Treemap view showing all GO terms, with the colors representing the enriched terms. If the term ratio is higher for the study set than the population set, the term is colored rose; otherwise, the term is colored blue. (C) The treemap is filtered to display carbohydrate metabolic process and all of this term’s children. (D) The GO Tree Navigator view can aid the user in navigating the GO tree hierarchy. All of the views are coordinated with one another such that selection in one view is propagated to all others.

The *Brucellae* are gram-negative, intracellular pathogens with the ability to infect multiple hosts. Individual *Brucella* species tend to have a host preference, but can be infectious to other species. *Brucella* infection, or brucellosis, causes undulant fever in humans and can be fatal if untreated. [25], [26]. The Centers for Disease Control and Prevention have classified *B. melitensis*, *B. suis*, and *B. abortus* as potential bioweapons due to their ability to easily infect humans. Brucellosis can also have severe economic effects on agriculture when livestock infections result in infertility, fetal loss, and reduced milk production. The *Brucella* genus has classically been described as containing six species, identified through their distinct host preferences and biotyping. *B. melitensis* infects goats and sheep, *B. abortus* infects cattle, *B. suis* infects pigs, *B. canis* infects dogs, *B. ovis* infects sheep, *B. neotomae* infects wood rats, and *B. microti* infects voles [27]. New species that infect marine mammals and have also been associated with human infection have recently been discovered. [28]. Currently, there are 12 completely sequenced *Brucella* strains available in public repositories with three strains representing *B. abortus*, one *B. canis* strain, four *B. melitensis* strains, one *B. ovis* strain, two *B. suis* strains, and one *B. microti* strain.

To identify genes or functions that could potentially be involved in either virulence or host preferences, the analyst must have the ability to group genomes together based on knowledge of the properties of each strain or species. A question-focused grouping of species, along with a query that supports the rational approach of comparing gene content in order to identify potential functional differences, automatically prompts exploration of potentially significant gene differentials when applied in GenoSets. In the current case studies we report that the visualizations provided pointers to gene families with known significance to function, as proof of concept. The same principle can be used in exploratory mode to identify gene targets from fresh data.

This data set includes a total of 38,436 CDS in the 12 genomes of which 24,762 have GO annotations. The coding regions are grouped into a total of 4,204 ortholog clusters.

### Case Study 1: Which Genes are Common among the Human Highly Pathogenic Strains of Brucella Spp.?

GenoSets allows the user to query presence or absence of genes in a genome, based on orthology, at multiple levels of the taxonomic hierarchy. When several genomes, for example different strains of a bacterial species, are aggregated at a higher taxonomic level, genes are classified as core, dispensable, and disparate, categorizations that may change at different levels of the taxonomy hierarchy. For example, the genes that are core to the *B. abortus* species are the genes found in all three of the *B. abortus* strains (strain 9-941, strain 2308, and strain S19). The *B. abortus* dispensable genes are found in some of the strains, but not all and the disparate are not found in any of the strains.

In Case Study 1, we are interested in genes that are common to strains that frequently cause infections in human hosts. *B. melitensis*, *B. suis*, and *B. abortus* species commonly infect humans, while *B. ovis* and *B. canis* strains are considered to be of low pathogenicity for humans [27]. A query was constructed in the Parallel Sets view for selecting the set of genes that do not have an ortholog in *B. ovis* or *B. canis* species, but are common to the high pathogenic species. Because some of the other species in this set include naturally attenuated or vaccine strains (*B. abortus* S19 and *B. melitensis* M5-90), this was a query designed to identify genes that were important in the high-pathogenic strains but did not necessarily cause attenuation. There are a total of 60 genes that meet these criteria, of which 100% have GO annotations. Figure 3A shows the construction of this query in Parallel Sets, and resulting details of the gene enrichment analysis displayed in the Enrichment Details view (Figure 3B).

The GO term with the smallest p-value in this analysis is the GO term “external encapsulating structure part” (log_10_(p) = −19). This category is associated with 20 genes that are grouped into two distinct ortholog clusters (Ortholog Cluster 100959 and 100911) (Figure 3C). One cluster associated with this GO term (Ortholog Cluster 100911) contains an OMP31-1 gene that encodes an outer membrane protein. Following up on this category, which we are prompted to explore because it emerges from the visualization, we find that the interface leads us to genes of possible biological significance. Several members of the Omp/Omp31 family are involved in virulence [29] and their use in vaccines against brucellosis has been suggested [30]. The omp genes are deleted from vaccine strain, B. melitensis Rev1. [31]. The type IV secretion system in *Brucella* (VirB) is integral in pathogenesis and has been shown to affect transcription of OMP31. More specifically, the inactivation of the virB operon was shown to decrease transcription of OMP 25/OMP31 and affects properties of the cell membrane in B. melitensis [32].

The other ortholog cluster (Ortholog Cluster 100959) associated with the GO term “external encapsulating structure part” contains genes annotated as branched-chain amino acid ATP-binding cassette (ABC) transporters (Figure 3B). Transporters are one means by which bacteria interact with their environment. Although a large portion of the *Brucella* genome consists of ABC systems, there is increasing evidence that ABC transporters are important in virulence and can be used as targets for vaccine development [33]. Because this specific transporter gene is not found in the low-pathogenicity strains, it may have a role in the degree of virulence or in survival in the host for the highly pathogenic strains.

The GO term “glycerol kinase activity” is also represented in this study set (log_10_(p) = −19). There is a single ortholog cluster associated with this category (Ortholog Cluster 100989– not shown). The cluster contains the erythritol kinase, eryA, meaning that it is overrepresented in the highly pathogenic strains. Erythritol metabolism has been associated with the ability by *Brucella* to cause abortions in livestock due to the levels of erythritol in the placenta of these animals [34]. The erythritol catabolic operon consists of four genes, eryA, eryB, eryC, and eryD. In the study set, EryA appears in both highly pathogenic species, even in the attenuated strains; however, further investigation into this operon revealed that the operon is disrupted in low-pathogenic species *B. ovis*, *B. canis*, and the naturally attenuated vaccine strain *B. abortus* S19. The *B. ovis* and *B. canis* are both missing the EryA genes and *B. ovis* is also missing the EryD gene in this operon. The vaccine strain contains a deletion that disrupts the coding regions of eryC and eryD (Crasta 2008).

### Case Study 2: Analysis of Host Preferences

To identify genes that are variable among the species, a query was built in Parallel Sets to compare gene content broadly among *B. suis*, *B. ovis*, *B. canis*, *B. melitensis*, and *B. abortus* (Figure 4). From the Parallel Sets display, a large set emerged in the visualization. Following the visual cues in the interface to explore this set highlights the utility of the system. Although not biologically obvious this gene set stands out because it is much larger than many of the other of the gene sets (with the exception of the set of genes that are core to all of the species studied). The set consists of genes that are not present in *B. ovis,* in the core genome of *B. suis* and *B*. *canis* species, but dispensable among the other species. This choice of criteria might not be immediately obvious, but when comparing the species in a broad context because this subset stands out visually we are prompted to explore its biological significance. Further interrogation of the GO terms associated with this set identifies numerous enriched GO terms such as uronic acid metabolic processes and transposase activity. The GO Treemap and GO Tree Navigator in Figure 4 show the overall GO enrichment for this study set.

The subset of genes in the uronic acid metabolic process contains a single ortholog cluster. This cluster consists of genes annotated as glucuronate/uronate isomerases (uxaC). This enzyme is part of the D-glucuronate degradation pathway used by a few bacterial species to metabolize glucuronate as the sole source of carbon. The generic glucuronate degradation pathway involves four genes: uxaC, uxuB, uxuA, and kdgK. Many of the organisms in this set have authentic frame-shift mutations in one or several of the genes required for this pathway. The *B*. *melitensis* strain ATCC 23457 has mutations in genes uxaC and uxaA and is the only *B*. *melitensis* species that does not have all the genes required for this pathway. The only *B. abortus* species that contains all the genes for this pathway is *B. abortus* S19 vaccine strain, while other abortus strains have lost the uxaC gene. The *B. ovis* strain ATCC 25840 has also lost the uxaC gene. For both the single representatives of their species, *B. canis* and *B. microti* both appear to have the entire pathway intact. Both *B. suis* strains contain the uxaC gene but have lost functions of the uxuB.

For comparison, a similar query was created by changing a single category from the query above. The previous query included genes that were not in *B. ovis* and for this comparison, we modified the query to include genes that were core to *B. ovis* and kept all other category values the same. The most enriched GO category for this study set was “enterobactin synthetase complex” (log_10_(p) = −25). The single ortholog cluster associated with this term contains genes for gene entD, a homolog of the gene in *Vibrio cholera* that is required for vibriobactin biosynthesis. The catechol siderophore, vibriobactin is a means by which many bacterial pathogens acquire the iron required for growth and survival [35]. A protective response in the host is limiting the amount of iron available to the bacteria. The restriction of iron is more important for intracellular pathogens, such as *Brucella spp*. because macrophages have developed the ability to reduce cytoplasmic iron [36]. An intact 2,3-dihydroxybenzioc acid (2,3-DHBA) sidespore produced by the entCEBA genes has been shown to be important in *Brucella* survival and infection especially in the presence of erythritol; A *Brucella* 2,3-DHBA sidespore mutant did not lose virulence in mouse model but a loss of virulence was observed in bovine model, probably due to the abundance of erythritol in bovine placenta [37].

The entD, and entF are homologous to the *E*. *coli* genes flank the entCEBA operon and it is speculated that these genes have the ability to form complex sidespores although their specific functions still need to be experimentally verified [36]. Analysis of the entF mutant showed significant growth inhibition in an iron limited environment and cell death for the mutant in the iron-limited conditions in the presence of erythritol [36]. Our genomic analysis of the entCEBA operon in the *Brucella spp*. presented here reveals *B*. *canis* and *B. ovis* have one of the four genes disrupted. However, they both appear to have an intact entD gene. The strains that do not have the entD gene are the *B. abortus* 9-9941 and *B. melitensis* ATCC 23457 strains. Overall, the strains that appear to have the intact entCEBA operon, entF, entD, and the erythritol operon are *B. abortus* 2308, *B. melitensis* 16M, M28, M5-90, *B. microti*, and *B. suis* 1330.

## Conclusions

We have developed the GenoSets software system for visual analysis of comparative genomics data. GenoSets uses a coordinated multiple view approach to enable set-based querying across data collections containing multiple genomes. The interface provides views that divide the genome content into sets based on properties or on membership in functional category hierarchies. GenoSets offers insight into features of interest by providing an analysis platform to visualize and explore complex queries. The design of the system is modular and many data types can be supported. The system simplifies the process of storing and integrating data and the visual interface supports the interactive development of complex queries.

Using the *Brucella* genus as a case study, we explore several ways the system can be used to construct genes sets that span multiple genomes, and show that the system can be used to successfully identify key features and functions of interest.

### Availability

GenoSets is release under the GNU General Public License (GPLv2). Installers for Windows, Mac, and Linux operating systems and the data used for the case studies are available at http://genosets.uncc.edu. The source code can be downloaded from http://code.google.com/p/genosets/.

## Supporting Information

Table S1 **EMBL Accession Information.** This file contains EMBL accession numbers and date of accession version for each of the genomes used in this analysis.
(DOCX)
