# Supplementary material for: GenoSets: Visual Analytic Methods for Comparative Genomics
Source: PLoS One. 2012 Oct 3;7(10):e46401. doi: 10.1371/journal.pone.0046401 (PMC3463605; doi:10.1371/journal.pone.0046401)
Supplement: Table S1 — EMBL Accession Information. This file contains EMBL accession numbers and date of accession version for each of the genomes used in this analysis. (DOCX) [file pone.0046401.s001.docx]

| **Species** | **Chromosome** | **Accession** | **Version Date** |
| --- | --- | --- | --- |
| Brucella abortus (strain 2308) | chromosome I | AM040264.1 | 2/19/2010 |
| Brucella abortus (strain 2308) | chromosome II | AM040265.1 | 2/20/2010 |
| Brucella abortus (strain S19) | chromosome 1 | CP000887.1 | 2/25/2010 |
| Brucella abortus (strain S19) | chromosome 2 | CP000888.1 | 2/26/2010 |
| Brucella abortus bv. 1 str. 9-941 | chromosome I | AE017223.1 | 2/17/2010 |
| Brucella abortus bv. 1 str. 9-941 | chromosome II | AE017224.1 | 2/18/2010 |
| Brucella canis (strain ATCC 23365 / NCTC 10854) | chromosome I | CP000872.1 | 2/23/2010 |
| Brucella canis (strain ATCC 23365 / NCTC 10854) | chromosome II | CP000873.1 | 2/24/2010 |
| Brucella melitensis biotype 2 (strain ATCC 23457) | chromosome I | CP001488.1 | 3/1/2010 |
| Brucella melitensis biotype 2 (strain ATCC 23457) | chromosome II | CP001489.1 | 3/2/2010 |
| Brucella melitensis bv. 1 str. 16M | chromosome I | AE008917.1 | 2/13/2010 |
| Brucella melitensis bv. 1 str. 16M | chromosome II | AE008918.1 | 2/14/2010 |
| Brucella melitensis M28 | chromosome 1 | CP002459.1 | 3/7/2010 |
| Brucella melitensis M28 | chromosome 2 | CP002460.1 | 3/8/2010 |
| Brucella melitensis M5-90 | chromosome I | CP001851.1 | 3/5/2010 |
| Brucella melitensis M5-90 | chromosome II | CP001852.1 | 3/6/2010 |
| Brucella microti (strain CCM 4915) | chromosome 1 | CP001578.1 | 3/3/2010 |
| Brucella microti (strain CCM 4915) | chromosome 2 | CP001579.1 | 3/4/2010 |
| Brucella ovis (strain ATCC 25840 / 63/290 / NCTC 10512) | chromosome I | CP000708.1 | 2/21/2010 |
| Brucella ovis (strain ATCC 25840 / 63/290 / NCTC 10512) | chromosome II | CP000709.1 | 2/22/2010 |
| Brucella suis (strain ATCC 23445 / NCTC 10510) | chromosome I | CP000911.1 | 2/27/2010 |
| Brucella suis (strain ATCC 23445 / NCTC 10510) | chromosome II | CP000912.1 | 2/28/2010 |
| Brucella suis 1330 | chromosome I | AE014291.4 | 2/15/2010 |
| Brucella suis 1330 | chromosome II | AE014292.2 | 2/16/2010 |

## Supplementary Table 1
